# Dark Flow, Depression and Multiline Slot Machine Play

**DOI:** 10.1007/s10899-017-9695-1

**Published:** 2017-06-06

**Authors:** Mike J. Dixon, Madison Stange, Chanel J. Larche, Candice Graydon, Jonathan A. Fugelsang, Kevin A. Harrigan

**Affiliations:** 1 Department of Psychology, University of Waterloo, Waterloo, ON Canada; 2 Gambling Research Lab, University of Waterloo, Waterloo, ON Canada

**Keywords:** Slot machines, Problem gambling, Dark flow, Depression

## Abstract

Multiline slot machines allow for a unique outcome type referred to as a loss disguised as a win (LDW). An LDW occurs when a player gains credits on a spin, but fewer credits than their original wager (e.g. 15-cent gain on a 20-cent wager). These outcomes alter the gambler’s play experience by providing frequent, albeit smaller, credit gains throughout a playing session that are in fact net losses. Despite this negative overall value, research has shown that players physiologically respond to LDWs as if they are wins, not losses. These outcomes also create a “smoother” experience for the player that seems to promote a highly absorbing, flow-like state that we have called “dark flow”. Past research has indicated that there may be a relationship between problem gambling status and dark flow, as well as between dark flow, depression, and gambling expectancies. In this study, we sought to further understand these relationships, while examining the influence of LDWs on game preference in the context of single versus multiline slots play. We used a realistic slot machine simulator equipped with a force transducer to measure how hard players pressed the spin button following different outcomes. This measure of arousal showed that LDWs were treated similarly to small wins. Participants overwhelmingly preferred the multiline game and experienced more positive affect while playing it, compared to the single-line game. Problem gambling severity index scores were related to dark flow in both games, but this relationship was stronger for the multiline game. Additionally, depression symptomatology and dark flow were strongly correlated in the multiline game, with significant relationships between depression and gambling expectancy, and gambling expectancy and dark flow ratings also emerging.

## Introduction

Slot machines are one of, if not the most, pervasive forms of casino gambling in North America (Schüll 2012). Ontario alone is home to over 22,000 individual slot machines (Ontario Lottery and Gaming Corporation [OLG] 2016). Despite their popularity, research has identified slot machines as a particularly dangerous form of gambling, as these machines harbour specific structural features capable of promoting problematic play for some (Griffiths 1993). While traditional 3-reel machines typically only allow players to wager on a single payline, newer multiline slot machines, as their name suggests, allow the player to wager on multiple paylines simultaneously—sometimes upwards of 20 lines at once. Not only do these multiline games allow for larger and more variable bet sizes throughout a playing session, but they also allow for a unique type of spin outcome called a *loss disguised as a win* (LDW; Dixon et al. 2010). An LDW occurs when a player gains credits, but the number of credits gained is *less* than the original bet on that spin. For example, if a player plays a 1-cent machine and bets 1 cent on each of 20 paylines (for a total spin bet of 20 cents) and gains back 15 cents on the spin, the player has actually experienced a net loss of 5 cents. The reason why we refer to these outcomes as losses *disguised* as wins is that despite being losing outcomes, the machine celebrates these outcomes with reinforcing sounds and animations that are quite similar to those that occur when a player wins back more than they wager (i.e., a gain of more than the 20 cent wager in the above example). In other words, the loss is disguised by the celebrations produced by the machine. On a physiological level, players respond to LDWs with increased arousal, as evidenced by heightened skin conductance levels (SCLs) compared to regular losses (Dixon et al. 2010). In a study by Dixon et al. (2010), skin conductance responses were equivalent between LDWs and true wins suggesting that novice players may have miscategorized LDWs as actual wins. The heightened arousal produced by these losing outcomes is problematic, given that arousal is considered to be a primary reinforcer of gambling behavior (Brown 1986). Taken together, the presence of LDWs in multiline slot machines makes it possible for players to be reinforced for spins on which they are losing money, enabling the inevitable mounting losses that a player will experience to be effectively masked as positive experiences.

In the Dixon et al. (2010) study, the finding that LDWs triggered heightened arousal signatures that were similar to true wins was based on data collected on novice players as they played a commercially available slot machine. As the participants received outcomes chosen by the machine’s random number generator, the exact number of full losses, true wins and LDWs experienced varied from player to player. It would be important to conceptually replicate the finding of similar reactions to wins and LDWs in a more controlled fashion. Furthermore, one might argue that experienced slots players (unlike the novice players tested in that study) would be less likely to react to LDWs and true wins in a similar fashion. Finally, a drawback of analyzing skin conductance responses during slots play is that participants must be instructed not to move the hand to which electrodes are attached—a situation which may prevent experienced gamblers from playing the game in a naturalistic fashion.

One promising, less invasive measure of arousal involves recording the force with which players initiate spins following different outcomes. In recent studies (Dixon et al. 2015, 2017), we showed that following losing outcomes, players initiated spins with minimal force. Following highly salient *actual* wins, the excitement and arousal of the win appeared to cause players to initiate the next spin with greater force. In the Dixon et al. (2017) study, there appeared to be a specific titration of force and win size—the bigger the win, the larger the force applied to initiate the next spin. Relative to skin conductance and its accompanying wires and movement restrictions, measuring the force applied to the spin button allows for more naturalistic play since the spin button is an intrinsic part of the game. In these initial studies, the slot machine simulator was shown on a laptop computer, and the force transducer was attached to a modified, mounted mouse button. In the current study we sought to replicate our force findings using a far more realistic simulator of slot machine play. We housed our simulator inside the cabinet of an actual slot machine that included the buttons to which frequent players were accustomed. By attaching the force transducer underneath the spin button so that it was invisible to the participant we were able to gather force data in a highly naturalistic play scenario—gamblers played what looked like an actual slot machine, interacting with it in the way they were used to, using an actual spin button. One goal was to use this realistic simulator to assess whether following LDWs, the force applied by players to initiate the next spin would be more similar to the force they applied following wins or following full losses. A pattern of minimal force for losses, and equivalent force for LDWs and small wins would conceptually replicate our previous findings using skin conductance responses that led us to believe that players were miscategorizing LDWs as wins rather than correctly categorizing them as losses.

Both traditional single-line games and modern multiline games are programmed to achieve a specific payback percentage (the percentage of wagers returned to the player). This payback percentage is typically between 85 and 98%, but is always less than 100% (allowing casinos to make a profit). When playing games that consist of a single payline there are no LDWs. Spins are either full losses (where the machine goes into a state of quiet), or true wins accompanied by auditory and visual feedback. In these games players tend to experience long chains of losses interspersed by occasional wins. On average the win sizes in these games tend to be relatively large to offset the many full losses so that (over the long term) the combination of frequent losses and infrequent wins will meet the programmed payback percentage of the machine. This type of game entails a “choppy” experience for the player: long series of losses where the machine is in a state of quiet, with interspersed, relatively large exciting wins. By contrast, games with LDWs are thought to “smooth” the play experience of gamblers (Schüll 2012). By presenting some of the losing outcomes in the form of LDWs, losing streaks may be less noticeable since they contain reinforcing sights and sounds. Importantly, these multiline games can have exactly the same payback percentage as the single-line games, but because players are given some credits on LDWs (albeit less than the spin wager) the true wins can be smaller and still meet the programmed payback percentage. Thus, players tend to experience smaller but more frequent wins, less frequent full losses, and because of the presence of many LDWs, far more spins accompanied by celebratory reinforcement than in the single-line games with the same payback percentage. One offshoot of this “smoothing” of the game experience is that these multiline games seem to be the game of choice for gamblers playing commercially available slot machines (Livingstone et al. 2008; Templeton et al. 2015).

In a controlled study using a slot machine simulator (Dixon et al. 2014), we assessed players as they played two sessions of a game with the same theme and payback percentage. In one session they bet 1 cent on a single-line and played 250 spins (1 cent per spin for 250 spins). In a second session they bet 1 cent on each of 20 lines (20 cents per spin for 250 spins). Even though the payback percentages were programmed to be similar, after the sessions 94% of players indicated that they preferred the multiline game to the single-line game. Additionally, when participants were polled about their experiences immediately after each game (using the two “positive affect” items of the Game Experiences Questionnaire; IJsselsteijn et al. 2007) they reported greater positive affect while playing the 20-line game than the 1-line game. In this study however, it could not be ruled out that players simply preferred (and experienced greater positive affect) for the game where they wagered more money per spin. Thus, a goal of the current study was to see if the preference for multiline games would hold when the bet size per spin between the 20-line and 1-line games was equated.

Beyond their preferability, one concerning aspect of multiline games is that they appear to be especially absorbing to players with gambling-related pathology (Dixon et al. 2014; Templeton et al. 2015). Players describe what Schüll (2005) refers to as the slot machine “zone”, where everything outside of the slot machine game becomes irrelevant as the player becomes completely immersed in the game. Such all-encompassing absorption can be found in descriptions of flow states (Csikszentmihalyi 1992). Although flow states are often associated with positive activities, Csikszentmihalyi himself has warned that for some individuals there may be an addictive quality to the constant seeking of the flow state—“the self becomes captive of a certain kind of order, and is then unwilling to cope with the ambiguities of life” (Csikszentmihalyi 2002, p. 62). This quote is eerily reminiscent of the description of slots gamblers who gamble to escape the depressing realities of their day-to-day lives (Abbot and Volberg 1996; Getty et al. 2000). In recognition of the potential negative consequences of becoming absorbed in slot machine games (e.g., mounting losses as time imperceptibly passes and the seeking of this state as a form of escape) we have come to refer to this state as “dark flow” (a contraction of “the dark side of flow”—see Partington et al. 2009).

In a previous study where we showed an association between dark flow and problem gambling (Dixon et al. 2014) we measured dark flow with the two flow items on the brief “in-game” version of the Game Experiences Questionnaire (IJsselsteijn et al. 2007). We showed that those at high-risk for gambling problems preferentially endorsed these flow items (significantly more than low-risk or non-problem gamblers) but only for the 20-line game. Here we sought to replicate the association between dark flow and problem gambling status. To acquire more stable estimates of dark flow we used five flow items from the full Game Experiences Questionnaire (IJsselsteijn et al. 2007). A related goal was to see if we could replicate the finding that multiline games are more prone to inducing this dark flow state than single-line games.

Finally, we sought to assess whether there was a relation between depressive symptoms and dark flow, as this may be indicative of those who seek to gamble on slot machines as a means of escaping depression. Central to this idea is the fact that individuals experiencing depression may be prone to ruminating about distressing, negative life events (Nolen-Hoeksema et al. 1994). The continuous nature of slots play, and the accompanying intermittent reinforcement may curtail such rumination for these individuals. If this is indeed the case, then the dark flow experienced by some players may be the state where they find escape from these negative ruminations. To explore this relationship, we sought to establish whether there was a relation between depression symptoms and dark flow. This would in turn establish that for some, slots play is negatively reinforcing—it provides a relief from the negative affect that these players experience outside of the slots context. To bolster this interpretation we also assessed players’ expectancies about how gambling *should* impact their mood. We proposed that for those who find slots play negatively reinforcing, this should be reflected in their expectancies about gambling even before they play. These people should indicate that part of the reason that they gamble is to elevate their mood, and to reduce tension and stress.

To address these questions, we conducted an experiment at a local casino wherein participants played both a realistic 20-line and 1-line slot machine simulator housed within an actual slot machine cabinet and equipped with a force transducer underneath the spin button. We assessed the problem gambling status of participants, as well as dark flow and positive affect during game play in both single-line and multiline games. The main goals of the current study are to test the following hypotheses: (1) as indexed by force, LDWs will be reacted to as though they are wins, not losses, (2) players will experience greater positive affect and prefer multiline games over single-line games, (3) dark flow states will be preferentially achieved through multiline games, as opposed to single-line games, and (4) there will be an association between the propensity to experience dark flow states while playing slots and problem gambling status, as well as a relation between dark flow states, depression, and the propensity to gamble in the hope of elevating mood and relieving tension.

## Method

### Participants

Participants were recruited from the lobby of Casino Brantford in Brantford, Ontario, Canada, between the hours of 9:00 and 16:00. All participants were screened to ensure that they were 19 years of age or older (the legal age to play slot machines in Ontario), not in treatment for problem gambling, and played slot machines at least once a month (to ensure that they were regular gamblers). Additionally, participants were administered the PGSI at the time of prescreening to assess current level of gambling pathology. We recruited specific numbers of participants with varying levels of problem gambling symptomatology, resulting in a sample that ranged from non-problem gamblers, to problem gamblers. Including these prescreening criteria allowed us to gather a large sample of problem gamblers. However, because we were attempting to recruit a specific number of problem gamblers, this resulted in having to turn away non-problem gamblers in the later stages of data collection. One hundred and fifty-three participants volunteered for the study in exchange for a $25.00 Walmart gift card and any winnings that accrued on the slot machine game during the experiment. All participants were prescreened to ensure that they played slot machines at least once a month (i.e. were frequent slots players). The average age of the sample was 58.96 years (SD = 14.7), and the sample was predominantly male (91 males, 62 females).

### Instruments and Materials

#### Problem Gambling Severity Index (PGSI)

The PGSI is a subscale of the Canadian Problem Gambling Index (CPGI), and is a valid and reliable screening tool for gambling problems and gambling severity in the general population (Cronbach’s alpha of 0.84; Ferris and Wynne 2001).

#### Gambling Related Cognitions Scale (GRCS)

The GRCS is a 23-item instrument designed to assess the cognitions that players hold about their gambling (Raylu and Oei 2004). Although we administered all 23 items, of exclusive interest in this study were the items that comprised the gambling expectancy subscale. Players were asked to endorse on a 7-point Likert scale the degree to which they agreed with the following items: “Gambling makes me happier”, “Gambling makes things seem better”, “Gambling makes the future brighter”, and “Having a gamble helps reduce tension and stress”. Gambling expectancy scores were the sum of these four items. The GRCS has high levels of reliability (Cronbach’s alpha of 0.93 for the full scale, 0.87 for the Gambling Expectancy subscale), as well as concurrent and criterion-related validity (Raylu and Oei 2004).

#### Depression, Anxiety and Stress Scale (DASS21)

The DASS21 is a 21-item instrument designed to assess the negative emotional states related to depression, anxiety, and stress (Lovibond and Lovibond 1995). Although we administered all 21 items, we only analyzed the 7 items comprising the depression subscale (items such as “I felt that life was meaningless”, “I felt down-hearted and blue” were scored on a 0 [did not apply to me over the last week] to 3 [applied to me very much or most of the time over the past week] scale). Depression scores were calculated by summing the individual depression items. The full DASS-21 has demonstrated good reliability (as measured by Cronbach’s alpha, 0.93), as has the depression subscale (0.88; Henry and Crawford 2005). The DASS-21 also shows good convergent and discriminant validity (Henry and Grawford 2005).

#### Game Experiences Questionnaire (GEQ)

This scale was used to gauge player experience of the slot machine playing session. The original scale (33 item version, core module) was developed to assess various aspects of engagement with media: immersion, flow, competence, positive and negative affect, tension, and challenge (IJsselsteijn et al. 2007). We presented participants with a shortened version of this scale, focusing only on items that were relevant to a gambling context. From this shortened version, only the two subscales of flow and positive affect were analyzed. For (dark) flow the following five items were administered: “I was fully occupied with the game”, “I forgot everything around me”, “I lost track of time”, “I was deeply concentrated in the game”, and “I lost connection with the outside world”. For positive affect the following five items were administered: “I felt content”, “I thought it was fun”, “I felt happy”, “I felt good”, and “I enjoyed it”. For each item, the player endorsed one of the following alternatives: not at all, slightly, moderately, fairly, or extremely. Dark flow scores and positive affect scores were calculated by averaging the responses to the five relevant dark flow and positive affect items. The GEQ has demonstrated validity and reliability, with Cronbach’s alpha values ranging from 0.71 to 0.89 for the various subscales included (Poels et al. 2007).

#### Measure of Game Preference

To determine each participant’s game preference, the following item was administered: “Overall, which game did you prefer playing?” to which participants chose either the 1-line or 20-line game.

#### Slot Machine Games

Participants played a slot machine simulator (“Sands of Splendor”) designed to mimic multiline slots play. Participants played two versions of the game, one in which they bet on 20 pay lines (20-line game), and the other in which they bet on a single payline (1-line game). In the 20-line game, participants wagered 1 credit on 20 paylines of a 1 cent machine. In the 1-line game, participants wagered 4 credits on 1 payline of a 5 cent machine. These configurations ensured an equal bet size between both games (20 cent total bet per spin). The payback percentage of the two games were equated (92.4%). The reinforcement schedule was based on the programming documents of a commercially available slot machine. Consistent with these documents, the number and size of wins between the games was not identical. The average size of the wins in the single-line game was $3.55. The average size of the wins in the multiline game was $1.23. The outcome characteristics for each game are displayed in Table 1.

**Table 1:** Occurrences of each outcome type in the 1-line and 20-line games

Outcome type	Losses	LDWs	Small wins	Large wins
Money gained (cents)	0	1–19	21–80	≥100
20-line game	168	49	21	12
1-line game	237	0	2	11

*LDW*s Losses disguised as wins

#### Force

To measure the force with which participants utilized the spin button, we installed a force transducer hidden underneath the spin button in each slot machine cabinet. This allowed us to continuously and unobtrusively measure the amount of force a participant used to initiate each spin. We used an algorithm that first found the local maximum force at the time of each spin initiation and then defined a 1100 ms window around this peak. Our measure of force was the local maximum minus the local minimum within this window.

### Procedure

All participants gave informed written consent before participating in the study. The University of Waterloo’s Office of Research Ethics approved all procedures in the study (ORE 18931). Participants first completed the PGSI, demographic questions (gender, age), and an item assessing how often they played slot machines in the last 12 months (adapted from the full CPGI). Following this, participants completed the 21 items from the DASS-21 (Lovibond and Lovibond 1995) and the 23 items from the Gambling Related Cognitions Scale (GRCS; Raylu and Oei 2004). Participants then completed one of the two slot machine games with game order (20-line vs. 1-line) counterbalanced between participants. After completing 250 spins on the first game, participants completed the GEQ with regards to the game that they had just completed, and then moved on to the next version of the game, once again followed by the GEQ. Participants then completed the Cognitive Reflection Test (Frederick 2005) and gave estimates of how much time elapsed during each game and the number of winning spins that they experienced during each game. These items were administered for purposes peripheral to the present study and will not be considered here.

Participants were then remunerated (the balance of the slot machine after gameplay, which was $10.00 for all participants, and a $25 Walmart gift card) and given the opportunity to take responsible gambling materials.

## Results

Of the 153 gamblers who initially volunteered for the study, 1 dropped out prior to completing the PGSI. Upon examining the force data, it became clear that a minority of participants appeared to simply be trying to finish the experiment as quickly as possible. These participants simply “bounced” the spin button repetitively without attending to the outcomes that were being displayed. An algorithm was used to calculate the total number of button presses players made during the 500-spin session. Although the modal value was (as expected) 500 button presses, there was considerable variability among players (ranging from 500 to over 5000 button presses). The decision was made to eliminate these participants from all analyses based on their force data. We used a cutoff of button presses greater than 20% more than what was needed to trigger the 500 spins. Thus any player pressing the button more than 600 times during the 500 spin session was eliminated. This resulted in 15 players being removed from the analysis. One additional participant whose force data was not recorded due to a technical error was also removed. After excluding the above players we were left with a total sample of 136 individuals.

### Force

**Fig. 1:**
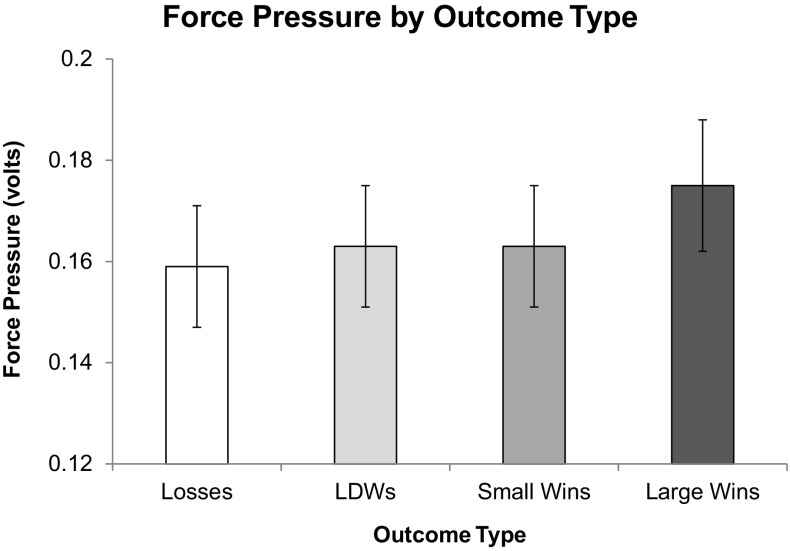
Mean force applied to the spin button following various outcome types. *Error bars* represent +1 SEM

The force with which players depressed the spin button following different outcomes in the multiline games was extracted using LabChart 7 software. Since our central predictions involved LDWs only the multiline game was analyzed (since single-line games do not have LDWs). The first and last spins that participants made were not analyzed (both losses). For each participant, the maximum amount of force applied to the spin button was determined following each outcome. All observations were sorted into bins based on the size of the credit gain that preceded this button press: losses (n = 166), LDWs (n = 49), small wins (20–80 credits; n = 21), and large wins (over 100 credits; n = 12). The force data was subjected to an outlier elimination procedure, wherein the criteria for rejection were adjusted based on the number of observations (Van Selst and Jolicoeur 1994). The average amount of force applied to the spin button following each type of outcome in the multiline game is displayed in Fig. 1. A repeated measures analysis of variance (with a Greenhouse-Geisser correction for sphericity) revealed a main effect of outcome type, *F*(2.088, 281.854) = 19.71, *p* < .001, partial eta squared = .127. Post-hoc analyses (Fisher’s LSD) indicated that more force was applied following LDWs compared to losses (*p* = .006), but that a similar level of force was applied following LDWs and small wins between 20 and 80 credits, (*p* = .89). Large wins of more than 100 credits elicited significantly greater amounts of force than any of the other outcome types (all *p*’s < .001).

### PGSI

Of the usable sample recruited (n = 136), 18 participants were classified as non-problem gamblers (PGSI score of 0), 49 as low risk (PGSI score of 1–4), 30 as moderate risk (PGSI score of 5–7), and 39 as problem gamblers (PGSI score of 8 or more).

### Game Preference and Positive Affect

In response to the game preference item, of the 136 usable participants 103 (75.74%) preferred the 20-line game to the 1-line game. In corroboration of these stated preferences, players gave significantly stronger positive affect endorsements for the 20-line game (*M* = 1.92) relative to the 1-line game (*M* = 1.69), *t*(135) = 4.08, *p* < .001, partial eta squared = .11. Positive affect scores were not correlated with PGSI scores for either the single-line game (*r*(134) = −.001, *p* = .995), or the multiline game (*r*(134) = .092, *p* = .286).

### Dark Flow

To assess which of the slot machine games encouraged a dark flow state, average flow scores from the five flow items of the GEQ were computed for each participant for each game type. Previous studies from our laboratory have indicated that individuals with greater problem gambling symptomology show an enhanced dark flow state in multiline games, as opposed to single-line games (Dixon et al. 2014). To replicate this result in a more realistic slot machine simulation environment, we computed correlations between PGSI scores and measures of dark flow for each game. Strong positive correlations were found between PGSI status and dark flow for both the 20-line game, *r*(134) = .572, *p* < .001, and the 1-line game, *r*(134) = .487, *p* < .001. These correlations were compared using Steiger’s test using Fisher’s r-to-z transformation (Steiger 1980). This comparison indicated a significantly larger dark flow/PGSI correlation in the 20-line game compared to the 1-line game, *z* = 2.03, *p* < .05.

To assess whether depression symptomatology was related to dark flow scores we conducted correlations between the depression subscale of the DASS21 and the dark flow scores in the (preferred) 20-line game. This analysis indicated a strong positive correlation, *r*(134) = .51, *p* < .001. Depression scores were also correlated with gambling expectancy scores, *r*(134) = .399, *p* < .001, and gambling expectancy scores were related to dark flow scores in the preferred game (*r*(134) = .439, *p* < .001).

## Discussion

Using the force applied to the spin button as a measure of players’ arousal, we showed that players applied the smallest amount of force following full losses, and large amounts of force following large wins. Crucially players applied the same amount of force following small wins and LDWs—a finding that conceptually replicates our previous findings using skin conductance responses (Dixon et al. 2010). This finding adds to a growing body of evidence that players psychologically miscategorize these outcomes as wins rather than losses. In a recent study (Dixon et al. 2014), we used post-reinforcement pauses (PRPs; the delays between the outcome delivery on a slot machine spin, and the initiation of the next spin) to show a similar finding. In that study, gamblers played a 20-line game where they bet 1 cent on each of 20 lines (20 cent bet) or a 1-line game (where they bet 1 cent on a single-line). The outcomes of greatest interest were those in which the machine gave back 2 cents to players. On the 1-line game this outcome represented a true win of 1 cent. On the 20-line game this outcome represented an LDW involving a net loss of 18 cents. The PRPs in both games were significantly longer than full losses. Crucially however, the PRPs for the true wins and the LDWs were similar in magnitude. Thus using SCRs (Dixon et al. 2010), PRPs (Dixon et al. 2014) or measures of force (the current study), the conclusions are the same—gamblers react to LDWs as though they are wins, not losses.

In the PRP study mentioned above we also asked players whether they preferred the 20-line game or the 1-line game. The vast majority of players (94%) preferred the 20-line game. Because of the way the study was designed we could not rule out that these preferences were driven by bet amounts (i.e., players simply preferred the game in which they risked more money). In the current study the bet amounts were equated (in the 20-line game they wagered 1 cent on each of twenty lines for a spin bet of 20 cents; in the 1-line game they wagered 20 cents on a single-line). The majority of players (76%) preferred the 20-line game to the 1-line game. This finding (where both bet amounts, and payback percentage across games were controlled) replicates the findings of Livingstone et al. (2008) who polled preferences of players playing on actual slot machines and showed that the majority of players preferred betting on the maximum number of lines available to them.

In our previous research (Dixon et al. 2014) we found that the 20-line game was particularly conducive to problem gamblers entering a state of dark flow. In that study, there was an interaction between gambling status and the game that was played. That is, those with more severe gambling problems became immersed in play, but only for the 20-line game, not for the 1-line game. As noted above, however, there were differences in the amount wagered on the two games. It may be that wagering only 1 cent per spin was not as engaging as wagering 20-cents per spin. Also the simulator was presented on a laptop with players using an external mouse to activate spins—a means of play very different from what they were used to. In the current research we tested players on a realistic simulator housed in an actual slot machine casing with the type of spin button they would be familiar with. We measured dark flow in each of the respective games using all five flow items from the GEQ (whereas the previous study used only 2 items). Perhaps most importantly, we equated the spin bet size in the 20-line and 1-line games. Using this more realistic simulator we showed that there were strong positive correlations between PGSI scores and dark flow, even for the 1-line game. Thus it may be that the failure to show any relation between gambling status and dark flow for the 1-line game in the previous study was because the amount wagered was so small, or the laptop simulator was so unlike an actual slot machine. Importantly, in the current study, although correlations between dark flow and PGSI scores were significant for both the 1-line and the 20-line games, the correlation between dark flow and problem gambling status was significantly more pronounced for the 20-line game. One interpretation of this finding is that it provides a conceptual replication of our previous work (Dixon et al. 2014) and suggests that those with gambling problems preferentially experience dark flow in 20-line games. In some ways when it comes to understanding the importance of the contribution of multiline play to the relationship between problem gambling and dark flow, the fact that players can experience dark flow during single-line play is made moot by the fact that the overwhelming majority of players prefer multiline games. That is, extending these results to actual casino gambling (outside of the experimental context used here) we can assume that most players will opt to play multiline games (Livingstone et al. 2008), and that it is the dark flow that they experience in the context of such play that is of primary importance. In other words, it may matter not how adept single-line games are capable of inducing dark flow, since the majority of players seldom play them.

Finally, we showed strong correlations between problem gambling scores on the PGSI and depression scores on the DASS. We interpret this correlation as being attributable to a subset of problem gamblers who gamble to escape depression. Our contention was that at least some depressed individuals are prone to ruminating about their problems in their day-to-day lives and that the continuous nature of slots play, and the inherent periodic yet unpredictable reinforcement that they experience while playing slots provides them with relief from such negative mentations. As some problem gamblers have told us—becoming immersed in slots is the *only* time they are not thinking about the sadness in their lives. In support of this notion, we found a significant relation between dark flow and depression, indicating that the dark flow state may be the place where some gamblers find this relief. Such relief may negatively reinforce their gambling behavior: these same participants who experience elevated dark flow scores also indicated that they expect gambling to elevate their mood and relieve tension.

In conclusion, LDWs may “smooth” the gamblers experience and provide a highly engrossing game experience characterized by frequent yet unpredictable reinforcement. Depressed individuals may ruminate about their problems and seek relief from such negative affect. The dark flow state they achieve whilst playing slots may provide them with the relief that they seek. This could explain not only why some gamblers expect that gambling will elevate their mood, but also explain the robust relations reported here among problem gambling, depression and the state of dark flow.
